# World Journal of Surgical Oncology reviewer acknowledgement 2014

**DOI:** 10.1186/s12957-015-0439-3

**Published:** 2015-02-27

**Authors:** Manoj Pandey, Thalyana Smith-Vikos

**Affiliations:** Bhopal Memorial Hospital and Research Center, Bhanpur, Bhopal, Madhya Pradesh India; BioMed Central, Floor 6, 236 Gray’s Inn Road, London, WC1X 8HB UK

## Abstract

The editors of *World Journal of Surgical Oncology* would like to thank all our reviewers who have contributed to the journal in Volume 12 (2014).

**Remzi Abali**

Turkey

**Mahmoud Abbas**

Germany

**Zakaria Abd Elmageed**

United States of America

**Essam Abdelalim**

Qatar

**Iman Abdelgawad**

Egypt

**Badr Abdull Gaffar**

United Arab Emirates

**Aswin George Abraham**

United Kingdom

**Pedro Acién**

Spain

**Pietro Addeo**

France

**Prasad Adusumilli**

United States of America

**Mehmet Agilli**

Turkey

**Sastha Ahanatha Pillai**

India

**Hanaa Ahmed**

Egypt

**Sung-Ja Ahn**

South Korea

**Masaki Aizawa**

Japan

**Sami Akbulut**

Turkey

**Alper Akcan**

Turkey

**Ali Akdemir**

Turkey

**Margarete Akens**

Canada

**Ali Akhavan**

Iran

**Javed Akhtar**

Pakistan

**Md. Zahid Akhter**

India

**Takashi Akiyoshi**

Japan

**Matthew Albert**

United States of America

**Manuel Alcántara Moral**

Spain

**Luca Antonio Aldrighetti**

Italy

**Abraham Alexander**

Canada

**George Alexiou**

Greece

**Abdullah Algharib**

United Kingdom

**Badar Alhadhrami**

Oman

**Bilal Al-Jiffry**

Saudi Arabia

**Eyas Alkhalili**

United States of America

**Marco Allaix**

Italy

**Asma AlNajjar**

Saudi Arabia

**Tahseen Al-Saleem**

United States of America

**Abdulmalik Altaf**

Saudi Arabia

**Giordano Alves**

Brazil

**Saadallah Al-Zacko**

Iraq

**Maria Raffaella Ambrosio**

Italy

**Amedeo Amedei**

Italy

**Soheila Aminimoghaddam**

Iran

**Michele Ammendola**

Italy

**M. Robyn Andersen**

United States of America

**Andreas Andreou**

Germany

**Chaisiri Angkurawaranon**

United Kingdom

**Nagaraju Anreddy**

United States of America

**Myron Arlen**

United States of America

**Paul Arnold**

United States of America

**Grazia Artioli**

Italy

**Andreas Artopoulos**

United Kingdom

**Christopher Aston**

United States of America

**Maurice Efana Asuquo**

Nigeria

**Sam Atallah**

United States of America

**Riccardo A. Audisio**

United Kingdom

**Pirvu Augustin**

France

**Ana Maria Autran**

Mexico

**Aïda Ayadi-Kaddour**

Tunisia

**Haruhuito Azuma**

Japan

**Woo Yong B Ae**

South Korea

**Hideo Baba**

Japan

**Mahwash Babar**

United Kingdom

**Sung Uk Bae**

South Korea

**Jae Hyun Bae**

South Korea

**Badrulhisham Bahadzor**

Malaysia

**Gopisankar Balaji**

India

**Andrzej Balcerowiak**

Poland

**Domingo Balderramo**

Argentina

**Elizabeth Baldini**

United States of America

**Mark Ball**

United States of America

**Jheelam Banerjee**

United States of America

**Tahmina Banu**

Bangladesh

**Nikolaos Barbetakis**

Greece

**John Barr**

United States of America

**Antonio Barrasa**

Spain

**Allison Barrett**

United States of America

**Stefano Basile**

Italy

**Stefano Basso**

Italy

**Rashmi Bathri**

India

**Marco Johannes Battista**

Germany

**Joel Baumgartner**

United States of America

**Daniel Baumunk**

Germany

**Daniel Becker**

United States of America

**Natalia Bejarano**

Spain

**Mehtap Beker Acay**

Turkey

**Seyda Belli**

Turkey

**Omar Bellorin**

United States of America

**Marcelo Beltran**

Chile

**Vladimír Benes**

Czech Republic

**Lothar Bergmann**

Germany

**Alejandro Bermudez-García**

Peru

**Emilio Bertani**

Italy

**Sergio Bertoglio**

Italy

**Luca Bertolaccini**

Italy

**Lorenzo Bianchi**

Italy

**Pierre Bigot**

France

**Vivek Bindal**

India

**Akwasi Boateng**

United States of America

**Francesco Boccardo**

Italy

**Giorgio Bogani**

Italy

**Martin Bohac**

Slovakia

**Ilka Boin**

Brazil

**Francesca Bolacchi**

Italy

**Eleni Bolanaki**

Greece

**Ronell Bologna-Molina**

Mexico

**Rodrigo Bonhin**

Brazil

**Nina Booken**

Germany

**Marco Borghesi**

Italy

**Marcus Bosenberg**

Brazil

**Jean-François Bosset**

France

**David Bostwick**

United States of America

**Konstantinos Boulas**

Greece

**Alaae Boutayeb**

Morocco

**Wilbur Bowne**

United States of America

**Federico Bozzetti**

Italy

**Riccardo Brachet Contul**

Italy

**Lars-Ove Brandenburg**

Germany

**Rachel Brennan**

United States of America

**Giuseppe Brisinda**

Italy

**William Brugge**

United States of America

**Joseph Buell**

United States of America

**Jakob Burcharth**

Denmark

**Franciszek Burdan**

Poland

**Mauricio Burotto**

United States of America

**Jonas Busch**

Germany

**John Byrn**

United States of America

**Daniela Cabibi**

Italy

**Neslihan Cabioglu**

Turkey

**Francisco Cabrera-Mendoza**

Mexico

**Xiang-Ran Cai**

China

**Ana Laura Calderón-Garcidueñas**

Mexico

**Pietro Caliandro**

Italy

**Armando Calogero**

Italy

**Juan Pablo Campana**

Argentina

**Luca Campana**

Italy

**Fábio Campos**

Brazil

**Ferdinando Carlo Maria Cananzi**

Italy

**Aras Emre Canda**

Turkey

**Alberto Candau-Alvarez**

Spain

**Myrna Candelaria**

Mexico

**Francisco Candido dos Reis**

Brazil

**Edwin Canete**

Philippines

**Robert Canter**

United States of America

**Hui Cao**

China

**Guangwen Cao**

China

**Bangwei Cao**

China

**Salvatore Cappabianca**

Italy

**Sergio Carandina**

France

**Philip Carrott**

United States of America

**William Carson**

United States of America

**Irina-Draga Caruntu**

Romania

**Marco Casaccia**

Italy

**Riccardo Casadei**

Italy

**Emanuele Castelli**

Italy

**Giorgio Cattoretti**

Italy

**Luigi Cavanna**

Italy

**Subrata Chakrabarti**

United States of America

**Sudipta Chakrabarti**

India

**Rubel Chakravarty**

India

**Stephen Chan**

Hong Kong

**Wen-Wei Chang**

Taiwan

**Benoit Chaput**

France

**Mary Charlton**

United States of America

**Mongkon Charoenpitakchai**

Thailand

**Grigoris Chatzimavroudis**

Greece

**Bicheng Chen**

China

**Zhe-Sheng Chen**

United States of America

**Kaiyun Chen**

China

**Xiaolei Chen**

China

**Jun Chen**

China

**Feng Chen**

China

**Yongshun Chen**

China

**Zong-You Chen**

China

**Wen Cheng**

China

**Wah Cheuk**

Hong Kong

**Pan Chi**

China

**Tawona Chinembiri**

South Africa

**Hung-Yi Chiou**

Taiwan

**Luis Chiva**

Spain

**Min Chul Choi**

South Korea

**Rubens Chojniak**

Brazil

**Siu Ho Chok**

Hong Kong

**Siu Ho Kenneth Chok**

China

**Tam-Lin Chow**

Hong Kong

**Shouvik Chowdhury**

India

**Fei Chu**

United States of America

**Sheng-Hua Chu**

China

**Saulius Cicenas**

Lithuania

**Cara Cipriano**

United States of America

**Shannon Clark**

United States of America

**Paul Clarkson**

Canada

**Fabricio Coelho**

Brazil

**Marcela Pecora Cohen**

Brazil

**Riccardo Colombo**

Italy

**Alfredo Conti**

Italy

**Gennaro Cormio**

Italy

**Murat Cosar**

Turkey

**Carlos E Costa Almeida**

Portugal

**Vitor Costa Simoes**

Portugal

**Lisa Coviello**

United States of America

**Immacolata Cozzolino**

Italy

**Carmen Criscitiello**

Italy

**Ion Cristobal**

Spain

**Guanglin Cui**

Norway

**Andrew Currie**

United Kingdom

**Glenda da Silva**

Brazil

**Rikke Dahlrot**

Denmark

**Juncheng Dai**

China

**Alberto Dal Molin**

Italy

**Rolando M. D'Angelillo**

Italy

**Karina Danilowicz**

Argentina

**Alexander Daoud**

United States of America

**Christopher Dardis**

United States of America

**Guenter Daxenbichler**

Austria

**Remco de Bree**

Netherlands

**Juan R. de los Toyos**

Spain

**Giuseppe De Luca**

Italy

**Tarek Debs**

France

**Emil Dediol**

Croatia

**Yvonne Dei-Adomakoh**

Ghana

**Michela Del Prete**

Italy

**Silvana Del Vecchio**

Italy

**Marcos Dellaretti**

Brazil

**Andreas K Demetriades**

United Kingdom

**Hakan Demirci**

Turkey

**Ilhan Demirci**

Germany

**Hatice Mirac Binnaz Demirkan**

Turkey

**Chadrick Denlinger**

United States of America

**Chadrick Denlinger**

United States of America

**Nunzio Denora**

Italy

**Kristoffer Derwinger**

Sweden

**Daniel Deschler**

United States of America

**Jasreman Dhillon**

United States of America

**Francesco Di Costanzo**

Italy

**Roberto Di Donato**

Italy

**Alberto Di Leo**

Italy

**Massimo Di Maio**

Italy

**Bernardo Dias Pereira**

Portugal

**Julio Diaz-Perez**

United States of America

**Andrew Dickinson**

Canada

**Claudius Diez**

Germany

**Dale Ding**

United States of America

**Xiangwu Ding**

China

**Marius Distler**

Germany

**Rosa Divella**

Italy

**Anthony Dixon**

United Kingdom

**Bozena Dobrzycka**

Poland

**Philipp Doebler**

Germany

**Xiaorong Dong**

China

**Zhou Donglei**

China

**Piotr Donizy**

Poland

**Efrat Dotan**

United States of America

**Dimitrios Dragoumis**

United Kingdom

**Lutao Du**

China

**Xiaohui Duan**

China

**Berna Bozkurt Duman**

Turkey

**Traian Dumitrascu**

Romania

**Edward Ebramzadeh**

United States of America

**Ovie Edafe**

United Kingdom

**Barish Edil**

United States of America

**Bijan Eghtesad**

United States of America

**Brian Egleston**

United States of America

**Günter Eisele**

Switzerland

**Christopher Ekwunife**

Nigeria

**Omar Elmalki**

Morocco

**Adel El-Naggar**

United States of America

**Ahmed Elzawawy**

Egypt

**Shigenobu Emoto**

Japan

**Giorgio Ercolani**

Italy

**Katerina Erokhina**

United States of America

**Emanuela Esposito**

Italy

**Jon Etxano**

Spain

**Laura Evangelista**

Italy

**Clarisse Eveno**

France

**René Fahrner**

Germany

**Mark Fairweather**

United States of America

**Khashayar Fakhrian**

Germany

**Nicolas Fakhry**

France

**Anna-Karin Falck**

Sweden

**Daniela Fanni**

Italy

**Walid Faraj**

Lebanon

**Sükösd Farkas**

Hungary

**Farshad Farshidfar**

Canada

**Giammarco Fava**

Italy

**Iain Feeley**

Ireland

**Yang Fei**

China

**Luigi Fenoglio**

Italy

**Clarissa Fernandes**

Brazil

**André Figueiredo**

Brazil

**Vittorio Fineschi**

Italy

**Enrico Fiori**

Italy

**Paul Fitzpatrick**

Sweden

**Rosario Fornaro**

Italy

**Dirk Forner**

Germany

**Simone Forterre**

Switzerland

**Lucio Fortunato**

Italy

**Caterina Fotia**

Italy

**Adam Enver Frampton**

United Kingdom

**Alessandro Franchello**

Italy

**Renato Franco**

Italy

**Marolleau François**

Belgium

**Richard K Freeman**

United States of America

**Deliang Fu**

China

**Takaaki Fujii**

Japan

**Shin Fujita**

Japan

**Takeo Fujita**

Japan

**Tetsuji Fujita**

Japan

**Tetsuo Fujita**

Japan

**Yoshinori Fujiwara**

Japan

**Yosuke Fukunaga**

Japan

**Naotake Funamizu**

Japan

**Philipp Funovics**

Austria

**Yoshitaka Furuya**

Japan

**Csaba Gajdos**

United States of America

**Valerio Gallotta**

Italy

**Andrea Galosi**

Italy

**Moses Galukande**

Uganda

**Sean Galvin**

Australia

**Donatella Gambini**

Italy

**Gerald Gantt, Jr.**

United States of America

**Rebeca Garcia-Roman**

Mexico

**Jerad Gardner**

United States of America

**Sue Garnett**

United Kingdom

**Louie Gaston**

United Kingdom

**Florian Gebauer**

Germany

**Georgios Georgiou**

Greece

**Hanspeter Gerber**

United States of America

**Sarbani Ghosh Laskar**

India

**Alex Giakoustidis**

United Kingdom

**Pierluigi Giampaolino**

Italy

**Alessandro Giardino**

Italy

**Joanna Gibson**

United States of America

**Anthony Gill**

Australia

**Erika Ginsburg**

United States of America

**Luca Giovanella**

Switzerland

**Sara Giovannoni**

Italy

**Pier Giulianotti**

United States of America

**Gabriel Glockzin**

Germany

**Paul Gobardhan**

Netherlands

**Sean Goh**

Australia

**Ilhami Gok**

Turkey

**Mónica Gomes**

Portugal

**Nelma Gomes**

United Kingdom

**Fernando Gómez**

Spain

**Li Gong**

China

**Weihua Gong**

China

**Luis González**

Spain

**David González-Flores**

Spain

**Philip Gordon**

Canada

**Mikel Gorostidi**

Spain

**Francois Gouin**

France

**Cara Govednik**

United States of America

**Claudia Gragnoli**

United States of America

**Ewen Griffiths**

United Kingdom

**Peter Grimminger**

Germany

**Valentina Grossi**

Italy

**Christina Gruber**

Austria

**Thomas Grunewald**

France

**Katharina Grupp**

Germany

**Salvatore Gruttadauria**

Italy

**Yan hong Gu**

China

**Guoli Gu**

China

**Wenxian Guan**

China

**Wenxian Guan**

China

**Maria Guembe**

Spain

**Francesco Guerra**

Italy

**Wang Guiqi**

China

**Dorothy Gujral**

United Kingdom

**Antonino Gulino**

Italy

**Serkan Guneyli**

Turkey

**Shutaro Gunji**

Japan

**Gang Guo**

China

**Lihe Guo**

China

**Yi Guo**

China

**Ketai Guo**

Germany

**Shailvi Gupta**

United States of America

**Simona Gurzu**

Romania

**David Gyorki**

Australia

**Alina Habic**

Romania

**Angelita Habr-Gama**

Brazil

**Vijay Hadda**

India

**Tetsuo Hagino**

Japan

**Oliver Hakenberg**

Germany

**Geoffrey Hallock**

United States of America

**Madoka Hamada**

Japan

**Elham Hamed**

Egypt

**Ho-Seong Han**

South Korea

**Ping Han**

China

**Christopher Hancock**

United States of America

**Ding-Jun Hao**

China

**Saroona Haroon**

Pakistan

**Yasuhisa Hasegawa**

Japan

**Ayshamgul Hasim**

China

**Eva Haspinger**

Italy

**Masaharu Hata**

Japan

**Etsuro Hatano**

Japan

**Ali Can Hatemi**

Turkey

**Peter Hauser**

Hungary

**Brian Hayes**

Ireland

**Mutlu Hayran**

Turkey

**Shoichi Hazama**

Japan

**Hong He**

Australia

**Ashraf Hefny**

Egypt

**Baukje Hemmes**

Netherlands

**Adam Hermanowicz**

Poland

**Adrian Hibberd**

Australia

**Shigeaki Higashiyama**

Japan

**Jens Hillengass**

Germany

**Tomoko Hirakata**

Japan

**Daisaku Hirano**

Japan

**Satoshi Hirano**

Japan

**Yosuke Hirasawa**

Japan

**Masahiko Hirota**

Japan

**Takeshi Hisa**

Japan

**Miguel Hissa**

Brazil

**Ming-Chih Ho**

Taiwan

**Thomas Höfner**

Germany

**Konstanze Holl-Ulrich**

Germany

**Hedieh Honarpisheh**

United States of America

**Simon Horenblas**

Netherlands

**Jun Horiguchi**

Japan

**Karoline Horisberger**

Germany

**Charlotte Höybye**

Sweden

**Bo-Yuan Hsiao**

Taiwan

**Chih-Cheng Hsieh**

Taiwan

**Meng Chiao Hsieh**

Taiwan

**Min Shu Hsieh**

Taiwan

**Chien-Jen Hsu**

Taiwan

**Jian-Kun Hu**

China

**Yingqi Hua**

China

**Dong Hua**

China

**Chao-Yuan Huang**

Taiwan

**Ling Huang**

China

**Jing Huang**

China

**Chao-Hung Hung**

Taiwan

**Soo Young Hur**

South Korea

**Sang Hwang**

Australia

**Maurizio Iacobone**

Italy

**Toni Ibrahim**

Italy

**Kenneth Iczkowski**

United States of America

**Benedetto Ielpo**

Spain

**Tsukasa Ikeura**

Japan

**Hisashi Ikoma**

Japan

**Kadri Ila**

Turkey

**Ray Iles**

United Kingdom

**Teruo Inamoto**

Japan

**Masafumi Inomata**

Japan

**Kentaro Inoue**

Japan

**Francesco Inzirillo**

Italy

**Orestis Ioannidis**

Greece

**Corey Iqbal**

United States of America

**Kamyar Iravani**

Iran

**Claudio Isella**

Italy

**Bjorn Isfoss**

Norway

**Hironori Ishihara**

Japan

**Takamichi Ishii**

Japan

**Arda Isik**

Turkey

**Fumiaki Isohashi**

Japan

**Yasuhiro Ito**

Japan

**Hideaki Ito**

Japan

**Shinji Itoh**

Japan

**Jakob Izbicki**

Germany

**Ira Jacobs**

United States of America

**Rana Jahanban-Esfahlan**

Iran

**Sumita Jain**

India

**James P Tam**

Singapore

**Fualal Jane Odubu**

Uganda

**Tae Jung Jang**

South Korea

**Tina Jaskoll**

United States of America

**Wojciech Jelski**

Poland

**Joon Jeong**

South Korea

**Hichem Jerraya**

Tunisia

**Bing JI**

China

**Shi-Wen Jiang**

United States of America

**Yong Jiang**

United States of America

**Chen Jin**

China

**Jie Jin**

China

**Tianbo Jin**

China

**Yinghua Jin**

China

**Jae-Won Joh**

South Korea

**Chris Jones**

United Kingdom

**Robin Jones**

United States of America

**Jean-Marc Joseph**

Switzerland

**Horng-Heng Juang**

Taiwan

**Mi Jun**

China

**Tareq Juratli**

Germany

**Vladimir Jurisic**

Serbia

**Elzbieta Kaczmarek**

Poland

**Ewa Kalinka-Warzocha**

Poland

**Kalpana Kalyanasundaram Bhanumathy**

Canada

**Erdinc Kamer**

Turkey

**Osamu Kamihira**

Japan

**Toshiya Kamiyama**

Japan

**De-Zhi Kang**

China

**Harsh Kanhere**

Australia

**Fatih Karadeniz**

South Korea

**Elias Karakas**

Germany

**Selda Karamil**

Turkey

**Thedoros Karantanos**

United States of America

**Bernd Kasper**

Germany

**Venkata Katabathina**

United States of America

**Rishil Kathawala**

United States of America

**Jaspreet Kaur**

India

**Shigeyuki Kawachi**

Japan

**Hisato Kawakami**

Japan

**Sakineh Kazemi Noureini**

Iran

**Mehmet Kefeli**

Turkey

**Edward Kendall**

Canada

**Mohammad Reza Keramati**

Iran

**Thomas Kerkhofs**

Netherlands

**Ugur KESICI**

Turkey

**Sonja Kessler**

Germany

**Akbar Khan**

Canada

**Hafiz Khan**

United States of America

**Piya Kiatisevi**

Thailand

**Yuko Kijima**

Japan

**Talat Kilic**

Turkey

**Min Joo Kim**

South Korea

**Jung Han Kim**

South Korea

**Hyun-Soo Kim**

South Korea

**Kyung Keun Kim**

South Korea

**Seong Hoon Kim**

South Korea

**Ji Eun Kim**

South Korea

**Soo Mi Kim**

South Korea

**Yasue Kimura**

Japan

**Johanna Kirchberg**

Germany

**Lawrence Kirschner**

United States of America

**Kinga Kiszka**

Poland

**Masahiro Kitada**

Japan

**Joji Kitayama**

Japan

**Jurgen Knuth**

Germany

**Shogo Kobayashi**

Japan

**Canan Kocaoglu**

Turkey

**Celalettin Kocaturk**

Turkey

**Bülent Koçer**

Turkey

**Ina Koch**

Germany

**Suman Kochhar**

India

**Takahiro Kogawa**

United States of America

**Naohiko Koide**

Japan

**Mahmut kuntay Kokanali**

Turkey

**Ender Koktekir**

Turkey

**Likurgos Kolilekas**

Greece

**Andrzej Komorowski**

Poland

**Tsunenori Kondo**

Japan

**Marina Kontogiorgi**

Greece

**Michael Kontos**

United Kingdom

**Piotr Korczynski**

Poland

**Ceren Korkmaz**

Turkey

**Ozge Korkmaz**

Turkey

**Ioannis Kostakis**

Greece

**Shin-ichi Kosugi**

Japan

**Christoforos Kotoulas**

Greece

**Andromachi Kougioumtzopoulou**

Greece

**Georgios Koutalellis**

Greece

**Mario Kramer**

Germany

**Avdyl Krasniqi**

Serbia

**Marco Krengli**

Italy

**Arvind Krishnamurthy**

India

**Naoshi Kubo**

Japan

**Naoshi Kubo**

Japan

**Harun Kucuk**

Turkey

**Ashok Kumar**

India

**Vinita Kumar**

India

**Vijay Kumar**

India

**Ramaiah Kumar**

India

**Yogesh Kumar**

United States of America

**Chikara Kunisaki**

Japan

**Jolanta Kupryjanczyk**

Poland

**Sedef Kuran**

Turkey

**Atsushi Kurata**

Japan

**Hiroaki Kuroda**

Japan

**Katarzyna Kusnierz**

Poland

**Aamet Kuvat**

Turkey

**Nisreen Kweider**

Germany

**Stefano La Rosa**

Italy

**Marco La Torre**

Italy

**Jean Lachowicz**

United States of America

**Chao-Han Lai**

Taiwan

**Jin-Yao Lai**

Taiwan

**Silvia Lai**

Italy

**Vinoth Kumar Lakshmanan**

India

**Matteo Landriscina**

Italy

**Brian Lang**

Hong Kong

**Cord Langner**

Austria

**Anders Christian Larsen**

Denmark

**Konstantinos Lasithiotakis**

Greece

**Johanna Laukkarinen**

Finland

**Daniela Lazar**

Romania

**Andreas Lazaris**

Greece

**Souhil Lebdai**

France

**Taek-Gu Lee**

South Korea

**Yoon-Suk Lee**

South Korea

**Andreas Leithner**

Austria

**Jose Leone**

United States of America

**Giovanni Leuzzi**

Italy

**Valerae Lewis**

United States of America

**Chung-Pin Li**

Taiwan

**Guoxin Li**

China

**Yi Li**

China

**Yong Li**

China

**Zhiyuan LI**

China

**Bin Li**

United States of America

**Jiangchao Li**

China

**Linfeng Li**

United States of America

**Yan Li**

China

**Yumin Li**

China

**Xinxiang Li**

China

**Zhenzhen Li**

China

**Enmin Li**

China

**Xiaoming Li**

China

**Du Lianfang**

China

**Libo Liang**

China

**Ting-bo Liang**

China

**Myong Cheol Lim**

South Korea

**Sey Kiat Lim**

Singapore

**Michael Lim**

United Kingdom

**Che Lin**

Taiwan

**Jules Lin**

United States of America

**Yuchang Lin**

China

**Daniele Lindemann**

Brazil

**Chen Ling**

United States of America

**Zhi-Qiang Ling**

China

**Marcelo Linhares**

Brazil

**Caigang Liu**

China

**Zhongchao Liu**

China

**Mu-Tai Liu**

Taiwan

**Min Liu**

China

**Fenglin Liu**

China

**Qingquan Liu**

China

**Qing-Xin Liu**

China

**Tang Liu**

China

**Zhenzhen Liu**

China

**Jing Liu**

China

**Song-Mei Liu**

China

**Zeng-Shan Liu**

China

**Lorenzo Livi**

Italy

**Raúl Loera Valencia**

Mexico

**Varut Lohsiriwat**

Thailand

**Tommaso Lombardi**

Switzerland

**Francesco Loria**

Italy

**Karen Lounsbury**

United States of America

**Peter Lovrics**

Canada

**Veronica Loy**

United States of America

**Xueguan Lu**

China

**Guillaume Luc**

France

**Giuseppe Lucarelli**

Italy

**Jian Luo**

China

**Xiaofeng Lv**

China

**Elena Maccaroni**

Italy

**João Machado-Neto**

Brazil

**Antonio Macrì**

Italy

**Annette Maczurek**

Australia

**Marcelo Madeira**

Brazil

**Yoshihiko Maehara**

Japan

**Gaetano Magro**

Italy

**Haider Mahdi**

United States of America

**Ashish Mahendra**

United Kingdom

**Aditya Maheshwari**

United States of America

**Haroon Majeed**

United Kingdom

**Tafadzwa Makarawo**

United States of America

**Masujiro Makita**

Japan

**Antti Mäkitie**

Finland

**Zahra Maleki**

United States of America

**Supriya Mallick**

India

**Veronika Mancikova**

Spain

**Antonio Manenti**

Italy

**Giuditta Mannelli**

Italy

**James Manson**

United Kingdom

**Nicoletta Maounis**

Greece

**Makia Marafie**

Kuwait

**Luigi Marano**

Italy

**Ilaria Marech**

Italy

**Francesco Saverio**

**Mari** Italy

**Luigi Mariani**

Italy

**Ugo Marone**

Italy

**Daniele Marrelli**

Italy

**Robert Martin**

United States of America

**Carlos Martinez**

Brazil

**Vicenç Martinez Vecina**

Andorra

**Wojciech Marusza**

Poland

**Satoshi Maruyama**

Japan

**Salvatore Andrea Mastrolia**

Israel

**Norikazu Masuda**

Japan

**Toshihiko Masui**

Japan

**Leandro Matos**

Brazil

**Paraskevi Matsota**

Greece

**Junichi Matsui**

Japan

**Akiyo Matsumoto**

Japan

**Kazumasa Matsumoto**

Japan

**Kazuhiro Matsumoto**

United States of America

**Yoshihiro Matsumoto**

Japan

**Hiroshi Matsushita**

Japan

**Tobias Mattei**

United States of America

**Tobias Mattei**

United States of America

**Susumu Matuskuma**

Japan

**Tobias Maurer**

Germany

**Andreas Mavrogenis**

Greece

**Yarrow McConnell**

Canada

**Mabula Mchembe**

Tanzania

**Jane McHowat**

United States of America

**Michael McIsaac**

Canada

**Fabio Medas**

Italy

**Fabio Medas**

Italy

**Aurora Medina-Sanson**

Mexico

**Meredith Medway**

Australia

**Diane Mege**

France

**Vinay Mehendale**

India

**Reza Mehrazin**

United States of America

**Jiong Mei**

China

**Mohamed Mekky**

Egypt

**Mostafa Melake**

Egypt

**Giulio Melloni**

Italy

**Muhammed Ashraf Memon**

Australia

**Fabio Mendes**

Brazil

**Fabrice Menegaux**

France

**Lourdes Mengual**

Spain

**Rudolf Mennigen**

Germany

**Francesca Merchionne**

Italy

**Walter Merkle**

Germany

**Roman Mezencev**

United States of America

**Nickolaos Michalopoulos**

Greece

**Anna Michno**

Poland

**Michal Mik**

Poland

**Milos Milosavljevic**

Serbia

**Ryogo Minamimoto**

Japan

**Bai Minghua**

China

**Olivier Mir**

France

**Vincenzo Mirone**

Italy

**Ali Mirsadeghi**

Iran

**Pradyumna Mishra**

India

**Koh Miura**

Japan

**Nobuyoshi MIyajima**

Japan

**Yasuyoshi Miyata**

Japan

**Toru Mizuguchi**

Japan

**Shugo Mizuno**

Japan

**Erito Mochiki**

Japan

**Sharon Moeno**

South Africa

**Rohit Moharil**

India

**Daniela Molena**

United States of America

**Adrian Molnar**

Romania

**Jennifer Monk**

Canada

**Katherine Moore**

Canada

**Luca Morelli**

Italy

**Mauricio Moreno**

United States of America

**Tasiuke Mori**

Japan

**Adriko Moses**

Uganda

**Mirna Mourtada-Maarabouni**

United Kingdom

**Dillip Muduly**

India

**Andre Mueller**

United States of America

**Oliver Mueller**

Germany

**Hetron Mweemba Munang'andu**

Norway

**Miguel Muñoz**

Spain

**Tomohiro Murakawa**

Japan

**Atsuhiko Murata**

Japan

**Adrian Murillo**

Spain

**Michael Murphy**

United States of America

**Domenico Murrone**

Italy

**Nora Mutalima**

United Kingdom

**Dattatraya Muzumdar**

India

**Ospan Mynbaev**

Russian Federation

**Karuppiah Nagaraj**

India

**Mitsutoshi Nakada**

Japan

**Naoki Nakagawa**

Japan

**Ryota Nakamura**

Japan

**Tomoki Nakamura**

Japan

**Satoshi Nakasu**

Japan

**Faina Nakhlis**

United States of America

**Tsutomu Namikawa**

Japan

**Nisana Namwat**

Thailand

**Moujhuri Nandi**

India

**Kannan Nar**

India

**Rajeswari Narayanappa**

India

**Masato Narita**

Japan

**Ofer Nativ**

Israel

**Pierina Navarria**

Italy

**Diana Navas Carrillo**

Spain

**Fred Nelson**

United States of America

**Barbara Netto**

Brazil

**Beng Kwang Ng**

Malaysia

**Rosa Nguyen**

United States of America

**Ourania Nicolatou-Galitis**

Greece

**Patricia Switten Nielsen**

Denmark

**Giuseppe Nigri**

Italy

**Yuji Nimura**

Japan

**Chunyu Niu**

China

**Xiaohui Niu**

China

**Takeo Nomi**

Japan

**Naohiro Nose**

Japan

**Markus Nottrott**

Germany

**Mohamed Nouh**

Egypt

**Alexander Novotny**

Germany

**Masahiro Nozawa**

Japan

**Achilleas Ntinas**

Greece

**Dimitrios Ntourakis**

France

**Ab Mutalib Nurul-Syakima**

Malaysia

**Daniel Nussbaum**

United States of America

**Chike Nzerue**

United States of America

**Toru Obuchi**

Japan

**Oliver O'Donovan**

United Kingdom

**Onder Ofluoglu**

United States of America

**Tomoko Ogawa**

Japan

**Yoshikazu Ogawa**

Japan

**Takao Ohtsuka**

Japan

**Hiroshi Okabe**

Japan

**Jiro Okami**

Japan

**Shinji Okano**

Japan

**Eiji Oki**

Japan

**Yoshinaga Okugawa**

Japan

**Scott Okuno**

United States of America

**Mana Oloomi**

Iran

**Georg Omlor**

Germany

**Ramesh Omranipour**

Iran

**Neamat Osman**

Egypt

**Naima Otmani**

Morocco

**Penelope Ottewell**

United Kingdom

**Anders Øverby**

Norway

**Jacqueline Oxenberg**

United States of America

**Seda Ozbek**

Turkey

**Irem Hicran Ozbudak**

Turkey

**Engin Ozcivici**

Turkey

**Salih Erpulat Ozis**

Turkey

**Safak Ozturk**

Turkey

**Furio Pacini**

Italy

**Somanath Padhi**

India

**Vincenzo Pagliarulo**

Italy

**Rama Pai**

United States of America

**Channing Paller**

United States of America

**Anshuman Pandey**

India

**Peter Panhofer**

Austria

**Yves Panis**

France

**Alberto Pansadoro**

Italy

**Salil Kumar Parida**

India

**Alexander Parikh**

United States of America

**Dhavan Parikh**

United States of America

**Do Joong Park**

South Korea

**Joon Seong Park**

South Korea

**In Hae Park**

South Korea

**Toshima Parris**

Sweden

**Dinah Parums**

United Kingdom

**Huillaume Passot**

France

**Vita Pasukoniene**

Lithuania

**Atish Patel**

United States of America

**Mayur Patel**

India

**Ramnik Patel**

United Kingdom

**Sapna Patel**

United States of America

**Byomokesh Patro**

India

**Kitty Pavlakis**

Greece

**Luigi Michele Pavone**

Italy

**Jennifer Payne**

Canada

**Irene Pecorella**

Italy

**Gong Peng**

China

**Guang Peng**

United States of America

**Hanwei Peng**

China

**Ruiyun Peng**

China

**Sharaf Karim Perdawood**

Denmark

**Daniel Perez**

Germany

**Carles Pericay**

Spain

**Sebastien Perreault**

Canada

**Hitesh Peshavariya**

Australia

**Laura Peterson**

United States of America

**Marco Petrillo**

Italy

**Federico Piccioni**

Italy

**Gaetano Piccolo**

Italy

**Eugene Pietzak**

United States of America

**Eugene Pietzak**

United States of America

**Alessandro Pileri**

Italy

**Antonio Pinto**

Italy

**Georgios Pitoulias**

Greece

**Jan Plock**

Switzerland

**Vlad Porumb**

Romania

**Ignasi Poves**

Spain

**Tomasz Powrózek**

Poland

**Heru Pradjatmo**

Indonesia

**Adriano Massimiliano Priola**

Italy

**Giuseppe Procopio**

Italy

**Georgios Psychogios**

Germany

**Shilpa Puli**

United States of America

**Thipachart Punyaratabandhu**

Thailand

**Sumyra Qadri**

India

**Shadi Qasem**

United States of America

**Aamer Qazi**

Canada

**Xing Qi**

China

**Zhan Qiang**

China

**Zhiqiang Qin**

United States of America

**Silvia Quadrelli**

Argentina

**Sajida Qureshi**

Pakistan

**Marius Raica**

Romania

**Janusz Rak**

Canada

**Estela Regina Ramos Figueira**

Brazil

**Suresh Rana**

United States of America

**Federica Rascio**

Italy

**Edward Ratovitski**

United States of America

**Deepak Rautray**

India

**John-David Rebibo**

France

**Rishindra Reddy**

United States of America

**Jeremy Rees**

United Kingdom

**Jean Marc Regimbeau**

France

**Moacyr Rêgo**

Brazil

**Chandrababu Rejeeth**

India

**Bharat Rekhi**

India

**Song Ren**

China

**Zefang Ren**

China

**Vivian Resende**

Brazil

**Juan Antonio Retamero Diaz**

Spain

**Lyndsay Rhodes**

United States of America

**Karl Riabowol**

Canada

**Riccardo Ricci**

Italy

**Carina Riediger**

Germany

**Luigi Rigacci**

Italy

**Philipp Riss**

Austria

**Rodolfo Rivas**

Mexico

**Cleo Robinson**

Australia

**Nathan Robison**

United States of America

**Jonathan Rodrigues**

United Kingdom

**Mario A. Rodriguez**

Mexico

**José Antonio Rodríguez-Montes**

Spain

**Jong-Lyel Roh**

South Korea

**Andrea Romano**

Italy

**Paul Rooney**

United Kingdom

**Giulio Rossi**

Italy

**Valentina Rossi**

Italy

**Rodnei Dennis Rossoni**

Brazil

**Krzysztof Roszkowski**

Poland

**Steven Rothenberg**

United States of America

**Kenneth Rothfield**

United States of America

**Giuseppe Rubini**

Italy

**Hannes Rudiger**

Switzerland

**Juan Ruiz-García**

Spain

**Maria Russell**

United States of America

**Antonio Russo**

Italy

**Giorgio Ivan Russo**

Italy

**Min-Hee Ryu**

South Korea

**Anton Sabashnikov**

United Kingdom

**Feridoun Sabzi**

Iran

**Lamiss Sad**

Egypt

**Tania Saibene**

Italy

**Leonard Saiegh**

Israel

**Akio Sakamoto**

Japan

**Alborz Salavati**

Iran

**Alejandro Salazar**

Spain

**Juan Antonio Salceda**

Argentina

**Mirna Saldívar**

Mexico

**Nikolaos Salemis**

Greece

**Milan Samardjiski**

Macedonia

**Paolo sammartino**

Italy

**Juan Sancho**

Spain

**Michele Santangelo**

Italy

**Matteo Santoni**

Italy

**Gouranga Santra**

India

**Akira Sasaki**

Japan

**Yusuke Sato**

Japan

**Caroline Saucier**

Canada

**Nadia Sawicka-Gutaj**

Poland

**Stefano Scabini**

Italy

**Peter Schemmer**

Germany

**Claus Schildberg**

Germany

**Maximilian Schmeding**

Germany

**Moritz Schmelzle**

Germany

**Carl Schmidt**

United States of America

**Maren Schulze**

Germany

**Roderich Schwarz**

United States of America

**Roman Sefr**

Czech Republic

**Masau Sekiguchi**

Japan

**Jayastu Senapati**

India

**Sanjeewa Seneviratne**

Sri Lanka

**Charalampos Seretis**

Greece

**Raffaele Serra**

Italy

**Sameh Sersar**

Egypt

**Monjri Shah**

United States of America

**Irshad Shaikh**

United Kingdom

**Guogen Shan**

United States of America

**Xiaobin Shang**

China

**Kiran Sharma**

Canada

**Daya Nand Sharma**

India

**Lizong Shen**

China

**Shun Shen**

China

**Victoria Shender**

Russian Federation

**Santosh Shenoy**

United States of America

**Gwo Tarng Sheu**

Taiwan

**Debing Shi**

China

**Jianhong Shi**

China

**Choong Nam Shim**

South Korea

**Kazuya Shinmura**

Japan

**Masahiro Shinoda**

Japan

**Akio Shiomi**

Japan

**Satoshi Shiono**

Japan

**Takkashi Shirobe**

Japan

**Vikas Sikri**

India

**Sabrina Silva**

Brazil

**Joao Manoel Silva Jr**

Brazil

**Nicola Silvestris**

Italy

**Nektaria Simiantonaki**

Germany

**Ronald Simon**

Germany

**Giuseppe Simone**

Italy

**Brijendra Singh**

India

**Cassian Sitaru**

Germany

**Ioannis Skandalos**

Greece

**Ayhan Sogut**

Turkey

**Leonardo Solaini**

Italy

**Olesya Solheim**

Norway

**Mamoon Solkar**

United Kingdom

**Chengli Song**

China

**Xicheng Song**

China

**Akshay Sood**

United States of America

**John Souglakos**

Greece

**Tutku Soyer**

Turkey

**Jens Sperling**

Germany

**Lucia Speroni**

United States of America

**Salvatore Squillaci**

Italy

**Anurag Srivastava**

India

**Katarzyna Starska**

Poland

**Philipp Storz-Pfennig**

Germany

**Russell Stothard**

United Kingdom

**Arne Streitbuerger**

Germany

**Philipp Ströbel**

Germany

**Moritz J Strowitzki**

Germany

**Tung-Hung Su**

Taiwan

**Takeki Sugimoto**

Japan

**Haruhiko Sugimura**

Japan

**Kiminori Sugino**

Japan

**Laurent Sulpice**

France

**Yang Sun**

United States of America

**Tong-Wen Sun**

China

**Wan-jun Sun**

China

**Simona Surdu**

United States of America

**Alexey Surov**

Germany

**Leslie Sutherland**

Canada

**Umesalma Syed**

India

**Isabella Syring**

Germany

**Marcella Szuecs**

Germany

**Sabina Tabaczar**

United Kingdom

**Alphonse Taghian**

United States of America

**Saeed Taheri**

Iran

**Wanyi Tai**

United States of America

**Takeshi Takahara**

Japan

**Tadao Takano**

Japan

**Shigeo Takebayashi**

Japan

**Mitsuko Takenaga**

Japan

**Kengo Takeuchi**

Japan

**Shigeyuki Tamura**

Japan

**Marcus Tan**

United States of America

**Lijie Tan**

China

**Yasuyuki Tanahashi**

Japan

**Kuniya Tanaka**

Japan

**Eiji Tanaka**

Japan

**Torgrim Tandstad**

Norway

**Kai-Fu Tang**

China

**Nobuhiko Tanigawa**

Japan

**Shinya Tanimura**

Japan

**Junko Tanizaki**

United States of America

**Ozgur Tanriverdi**

Turkey

**Tawee Tanvetyanon**

United States of America

**Yungan Tao**

France

**Syed Tariq**

United Kingdom

**Muhammad Tariq**

Pakistan

**Adriana Tavares**

Portugal

**Ahmed Tawfik**

Egypt

**Giovanni Domenico Tebala**

Isle of Man

**Mohamedtaki Tejani**

United States of America

**Onur Telli**

Turkey

**Chris Terhaard**

Netherlands

**Evangelos Terpos**

Greece

**Ugo Testa**

Italy

**Cagatay Tezel**

Turkey

**Serdar Tezelman**

Turkey

**Hardik Thakker**

India

**Binay Thakur**

Nepal

**Bridie Thompson**

Australia

**Henrik Thorlacius**

Sweden

**Ole Thorlacius-Ussing**

Denmark

**Shifu Tian**

United States of America

**Mei Tian**

China

**Yuanhu Tian**

China

**Yanmei Tie**

United States of America

**Xavier Tillou**

France

**Andreas Toepfer**

Germany

**Gabriele Toietta**

Italy

**Yuji Toiyama**

Japan

**Kazuaki Tokodai**

Japan

**Yukio Tokumitsu**

Japan

**Jeffrey Tomaszewski**

United States of America

**Erkan Topkan**

Turkey

**William Tormey**

Ireland

**Alfonso Torquati**

United States of America

**Valerie Touitou**

France

**Edward Tredget**

Canada

**Giorgio Treglia**

Switzerland

**Matthew Trendowski**

United States of America

**Anna Truini**

Italy

**Stavros Tryfon**

Greece

**Gary Tse**

Hong Kong

**Nikolaos Tsoukalas**

Greece

**Takayuki Tsuchiya**

United States of America

**Hiroyuki Tsuchiya**

Japan

**Masumi Tsujiwaki**

Japan

**Nelson Hirokazu Tsuno**

Japan

**Shigeru Tsunoda**

Japan

**Thomas Tu**

Australia

**Kangsheng Tu**

China

**Ibrahim Turker**

Turkey

**Tamara Tyrinova**

Russian Federation

**Hiroshi Uchinami**

Japan

**Alexis Ulrich**

Germany

**Akira Umemura**

Japan

**Michiaki Unno**

Japan

**Taizen Urahashi**

Japan

**Jalal Vahedian**

Iran

**J. Fernando Val-Bernal**

Spain

**Katherine Valeros**

Singapore

**Sniya Valsa sudhakar**

India

**Cornelius van de Velde**

Netherlands

**W.A. van der Zwan**

Netherlands

**Ruud van Erve**

Netherlands

**Thijs van Oudheusden**

Netherlands

**Petr Vavra**

Czech Republic

**Moniek Vedder**

Netherlands

**Paul Veenboer**

Netherlands

**Giovanni Vennarecci**

Italy

**Marilena Vered**

Israel

**Sebastien Vergez**

France

**Helene Verheije**

Netherlands

**Aysegul Verim**

Turkey

**Satyajeet Verma**

India

**Vaclav Vetvicka**

United States of America

**Rafael Vicens**

United States of America

**Ramon Vilallonga**

Spain

**Rene Villadsen**

Denmark

**José-Sebastián Villalón-López**

Mexico

**Alessandro Vitale**

Italy

**Marco Vitellaro**

Italy

**Dimitris Vlachodimitropoulos**

Greece

**My von Euler-Chelpin**

Denmark

**Christian von Falck**

Germany

**Anna Dorothea Wagner**

Switzerland

**Go Wakabayashi**

Japan

**Jennifer Waljee**

United States of America

**David Wallace**

United Kingdom

**Rohan Walvekar**

United States of America

**Wan Faiziah Wan Abdul Rahman**

Malaysia

**Jin Wang**

China

**Jianhua Wang**

China

**Kun Wang**

China

**Qifeng Wang**

China

**Meilin Wang**

China

**HaiTao Wang**

China

**Yan Wang**

China

**Yurong Wang**

China

**Binquan Wang**

China

**Zhong Wang**

China

**Blake Warner**

United States of America

**Satish Kumar Warrier**

Australia

**Jun Watanabe**

Japan

**Rafal Watrowski**

Germany

**Jianteng Wei**

China

**Helmut Weiss**

Austria

**Bernard Weissman**

United States of America

**Annet Westers-Attema**

Netherlands

**Phillip White**

United States of America

**Andreas Wicki**

Switzerland

**Lisa wiechmann**

United States of America

**Sean Williamson**

United States of America

**Timothy Wilson**

United States Minor Outlying Islands

**Andrzej Wincewicz**

Poland

**Alexander Winter**

Germany

**Christopher Wong**

Australia

**Gerald Wright**

United States of America

**Tao Wu**

China

**Chuanlong Wu**

China

**Jian Wu**

China

**Xinjiang Wu**

United States of America

**Bin Wu**

China

**Weiyun Wu**

China

**Jing Wu**

China

**Xudong Wu**

China

**Xing Zhong Wu**

China

**Jiazeng Xia**

China

**Wang Xiaoguang**

China

**Guangqin Xiu**

China

**Jiejie Xu**

China

**Fang Xu**

China

**Yongde Xu**

China

**Zhi-Xiang Xu**

United States of America

**Sun Xuecheng**

China

**Zhu Xulong**

China

**Daisuke Yabe**

Japan

**Zhang Yabing**

China

**Toshiki Yajima**

Japan

**Kazutaka Yamada**

Japan

**Sohsuke Yamada**

Japan

**Tomohiro Yamada**

Japan

**Takashi Yamada**

Japan

**Hidetaka Yamamoto**

Japan

**Kazuyoshi Yamamoto**

Japan

**Kazuhiro Yamanoi**

Japan

**Hiroharu Yamashita**

Japan

**Koji Yamashita**

Japan

**Ken Yamaura**

Japan

**Dongrong Yang**

China

**San-Nan Yang**

Taiwan

**Zhonghan Yang**

China

**Haihua Yang**

China

**Tomoyuki Yano**

Japan

**Masaahiko Yano**

Japan

**Katharine Yao**

United States of America

**Hongtao Ye**

United Kingdom

**Hui-Ling Yeh**

Taiwan

**Chun-Nan Yeh**

Taiwan

**Serkan Yener**

Turkey

**Fahri Yetisir**

Turkey

**Cheng-Har Yip**

Malaysia

**Mitsuhiro Yoneda**

United States of America

**Tuck Yean Yong**

Australia

**Zheng Yong**

China

**Zheng Yong**

China

**Zheng Yong**

China

**Kazuhiro Yoshida**

Japan

**Takaki Yoshikawa**

Japan

**Yasuo Yoshimura**

Japan

**Tateki Yoshino**

Japan

**Hironori Yoshiyama**

Japan

**Zongbing You**

United States of America

**Christopher Young**

Australia

**Qiujun Yu**

China

**Pengfei Yu**

China

**Zhi-Gang Yu**

China

**Wu Yu Jen**

Taiwan

**Zhong-Yu Yuan**

China

**Takeshi Yuasa**

Japan

**Sumbul Zaheer**

Singapore

**Nicola Zampieri**

Italy

**Massoumeh Zargaran**

Iran

**Nick Zavras**

Greece

**Bernhard Zelger**

Austria

**Alessandro Zerbi**

Italy

**Arife Zeybek**

Turkey

**Qiang Zhan**

China

**Xu Zhang**

China

**Chunlin Zhang**

China

**Hua Zhang**

United Kingdom

**Jianhua Zhang**

United States of America

**Jun Zhang**

China

**Xiu-Feng Zhang**

China

**Guo-xin Zhang**

China

**Qu Zhang**

United States of America

**Xiaoyu Zhang**

China

**Sen Zhang**

China

**Suzhan Zhang**

China

**Teng Zhang**

China

**Xing-Hua Zhang**

China

**Gang Zhao**

China

**Liang Zhao**

China

**Yu Pei Zhao**

China

**Hui Zhao**

China

**Jing Zhao**

United States of America

**Shuai Zhen**

China

**Liduan Zheng**

China

**Hua-chuan Zheng**

China

**Jian-yong Zheng**

China

**Yadong Zheng**

China

**Shusen Zheng**

China

**Song Zhengbo**

China

**Qinghua Zhou**

China

**Lijun Zhou**

China

**Li Zhou**

China

**Weidong Zhou**

United States of America

**Zhuwei Zhou**

China

**Tong Zhou**

China

**Shui-Hong**

**Zhou**

China

**Zhi-Xiang Zhou**

China

**Hua Zhu**

United States of America

**Xueqiong Zhu**

China

**Rafal Zielinski**

Poland

**Vincent Zimmer**

Germany

**Ouliana Ziouzenkova**

United States of America

**Panagiotis Zis**

Greece

**Carmine Zoccali**

Italy

**Xiangyun Zong**

China

**Murat Zor**

Turkey

**Massimiliano Zuccaro**

Italy

**Changjing Zuo**

China

**Li Zuo**

United States of America

